# All‐Aqueous Phase Segregation Integrated Electrochemical Aptamer Biosensor Enables Picomolar Detection of SARS‐CoV‐2 Spike Protein

**DOI:** 10.1002/smll.202503466

**Published:** 2025-10-03

**Authors:** Ryan Ho‐Ping Siu, Yage Zhang, Sihan Liu, Andrew Brian Kinghorn, Wei Guo, Ho Cheung Shum, Julian Alexander Tanner

**Affiliations:** ^1^ School of Biomedical Sciences Li Ka Shing Faculty of Medicine The University of Hong Kong Hong Kong SAR 999077 China; ^2^ Guangdong Key Laboratory of Biomedical Measurements and Ultrasound Imaging School of Biomedical Engineering Shenzhen University Medical School Shenzhen University Shenzhen 518060 China; ^3^ Department of Mechanical Engineering The University of Hong Kong Hong Kong SAR 999077 China; ^4^ Advanced Biomedical Instrumentation Centre Hong Kong Science Park Shatin, New Territories Hong Kong SAR 999077 China; ^5^ Department of Chemistry & Department of Biomedical Engineering City University of Hong Kong Hong Kong SAR 999077 China; ^6^ Materials Innovation Institute for Life Sciences and Energy (MILES) HKU‐SIRI Shenzhen Guangdong 518063 China; ^7^ School of Biomedical Engineering The University of Hong Kong Hong Kong SAR 999077 China

**Keywords:** aptamer, electrochemical biosensor, liquid‐liquid phase separation, SARS‐CoV‐2 spike protein

## Abstract

Electrochemical aptamer biosensors (E‐ABs) are promising tools for rapid point‐of‐care (POC) diagnosis which utilize aptamers for the molecular recognition of specific disease biomarkers. However, E‐AB sensitivity is typically limited by the binding affinity (*K_D_*) of the aptamer (often in the micromolar‐nanomolar range), while biomarker concentrations in biofluids are often at the picomolar level or below. Liquid‐liquid phase separation (LLPS) provides a robust framework to concentrate the target biomarker based on affinity‐controlled partitioning within an all‐aqueous environment. In this study, the integration of LLPS into E‐AB is reported to overcome the sensitivity limitation and achieve picomolar Severe acute respiratory syndroome coronavirus 2 spike protein (SARS‐CoV‐2 S protein) detection. Preferential spike protein partition upon LLPS is verified by microscopic imaging and biochemical assays. The LLPS‐integrated E‐AB is developed by a peroxidase catalytic reaction to generate electrical signals in the presence of the S protein. The LLPS demonstrates successful protein concentration from synthetic human biofluids and leads to at least 100‐fold sensitivity improvement to the picomolar level of the limit of detection (LOD) by altering the volume ratio (VR) of the two segregated phases. This is the first report of an LLPS‐embedded E‐AB application, advancing the sensitivity of aptamer electrochemical biosensing without a multi‐step downstream signal amplification cascade.

## Graphical abstract

A liquid‐liquid phase separation (LLPS) method is integrated into electrochemical aptamer biosensor (E‐AB) for improved sensitivity against SARS‐CoV‐2 spike protein. The LLPS successfully enriches spike protein prior to the E‐AB detection to achieve low picomolar limit of detection, applicable in synthetic human biofluids such as saliva and urine.

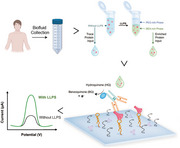

## 1 Introduction

Electrochemical aptamer biosensors (E‐ABs) detect specific biomarkers via molecular recognition mediated by single‐stranded DNA aptamers, transducing binding into electrical signals.^[^ ^1^, ^2^, ^3^ ^]^ Developing E‐AB over traditional methods like (Quantitative Reverse Transcription Polymerase Chain Reaction) RT‐qPCR or lateral flow immunoassay for infectious disease diagnosis is of great interest due to its high specificity, rapid response, cost‐effective device fabrication, and real‐time continuous measurement capabilities.^[^ ^4^, ^5^, ^6^ ^]^ These are crucial attributes for rapid and large‐scale community screening and/or diagnostics.

Although E‐ABs offer comparative advantages over their counterparts for epidemiological diagnosis, applications for both clinical and community testing are limited. The reason may be a combination of insufficient detection sensitivity and low biomarker concentrations in human biofluids.^[^ ^7^, ^8^ ^]^ The traditional aptamer selection process known as systematic evolution of ligands by exponential enrichment (SELEX) yields canonical aptamers that bind to their target at the nano to micromolar affinity in terms of the dissociation constant (*K_D_*). However, model biomarkers, such as the SARS‐CoV‐2 spike (S) protein found in human saliva, are present at the picomolar (pm) scale during early infection,^[^ ^9^ ^]^ far lower than the affinity of the identified aptamers.^[^ ^10^, ^11^, ^12^ ^]^ Therefore, existing E‐ABs may fall short in achieving the required clinical sensitivity.^[^ ^13^, ^14^ ^]^ Although specific studies have reported multimeric aptamer designs to improve the binding avidity against the S protein trimer,^[^ ^15^, ^16^, ^17^ ^]^ current research is focused on developing generalizable downstream signal amplification strategies to overcome the detection barrier when adopting the monomeric ssDNA aptamers. While the use of conductive nanomaterials, DNA nanotechnologies, or electrochemical transistors has enabled ultrasensitive detection,^[^ ^18^, ^19^, ^20^, ^21^, ^22^, ^23^ ^]^ the increased cost and assay complexity often do not favor (point‐of‐care) POC applications. Hence, a single‐step signal amplification approach to empower E‐AB as a rapid POC solution for future pandemic control is crucial.

In this study, we demonstrate a novel sample concentration mechanism called liquid‐liquid phase separation (LLPS) as an upstream signal amplification solution combined with E‐AB to achieve picomolar sensitivity for the Coronavirus disease 2019 (COVID‐19) S protein. Recent studies have shown that LLPS can trigger unique electrochemical effects crucial for cellular physiology,^[^ ^24^ ^]^ a concept that has yet to be translated into E‐AB assays. The LLPS is initiated from an aqueous solution consisting of two water‐soluble and biocompatible polymers, namely polyethylene glycol (PEG) and dextran (DEX).^[^ ^25^, ^26^ ^]^ When polymer concentrations surpass a critical threshold, spontaneous segregation into two aqueous phases occurs due to dominant polymer‐polymer interactions over mixing entropy.^[^ ^26^ ^]^ Each phase possesses distinct physicochemical properties and exhibits differential solvation energy for the S protein, prompting its preferential migration to a specific phase to minimize total free energy.^[^ ^27^, ^28^ ^]^ This partitioning behavior increases the local concentration of the S protein. Besides, the concentrated polymeric solution creates a macromolecular crowding environment resembling the intracellular matrix.^[^ ^29^, ^30^ ^]^ The presence of crowding reagents has been proven to enhance enzymatic performance and binding between ligands and analytes,^[^ ^31^, ^32^, ^33^ ^]^ further promoting the sensitivity of enzymatic E‐AB with improved target protein capturing efficiency. LLPS enriches protein biomarkers in 15 min while preserving biological activity for aptamer recognition.^[^ ^34^ ^]^ The partitioning of S protein into DEX‐rich fraction is confirmed through real‐time microscopic imaging and Enzyme‐Linked Oligonucleotide Assay (ELONA). A peroxidase‐based sandwich E‐AB utilizing aptamer SNAP1.50 is designed to quantify the S protein.^[^ ^12^ ^]^ Successful S protein enrichment improves E‐AB sensitivity by over 100‐fold and reaches a low pm limit of detection. While not commonly utilized in diagnostics, this study represents the first report to integrate LLPS into E‐AB and demonstrate advanced sensitivity for potential pandemic control.

## 2 Results

### 2.1 Real‐Time Microscopic Fluorescence Imaging of Spike Protein Partitioning

**Figure 1:**
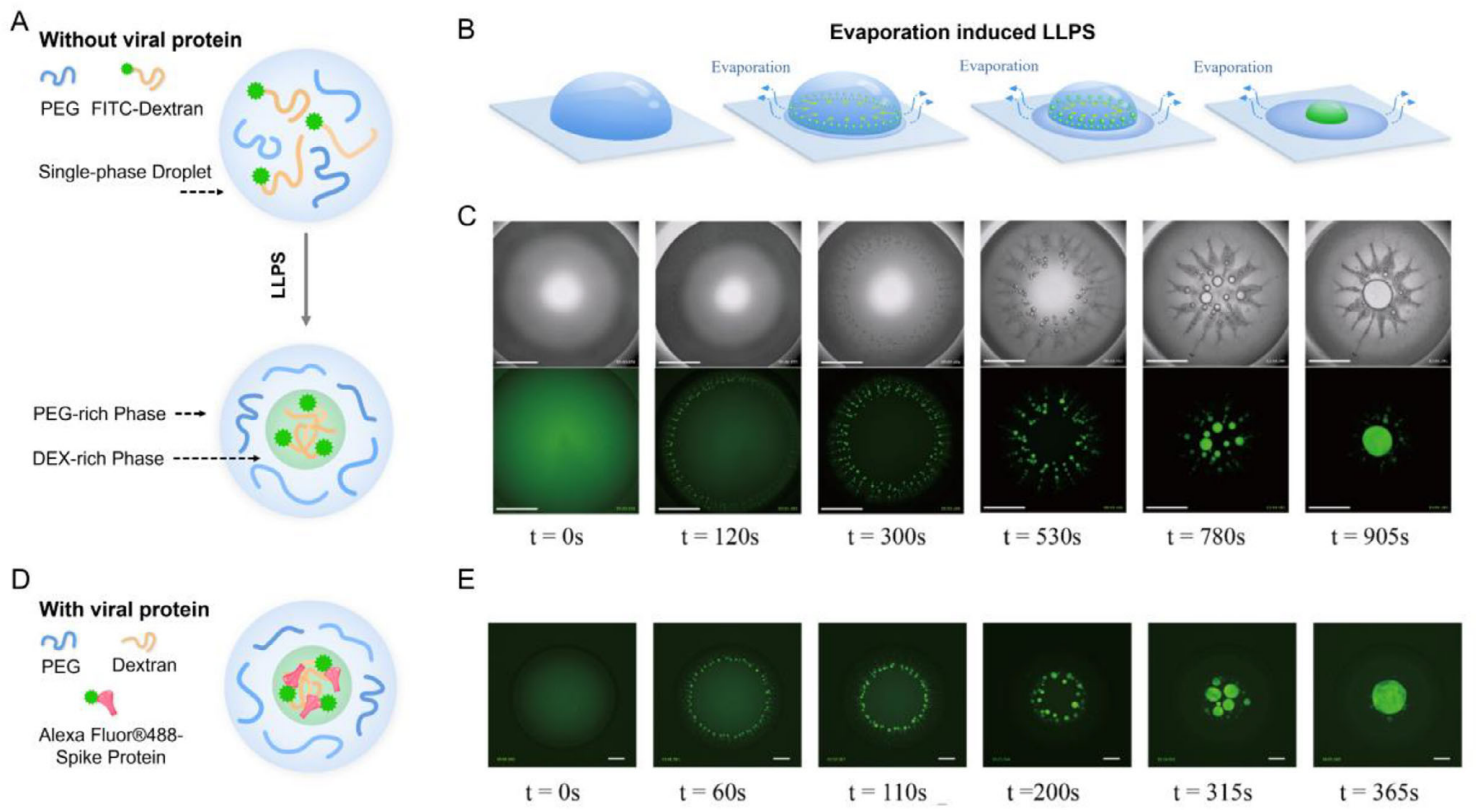
Evaporation‐induced spontaneous liquid‐liquid phase separation (LLPS) within a sessile droplet, where SARS‐CoV‐2 spike protein localizes into a dextran‐rich compartment over time. A) Schematic of phase separation inside an aqueous sessile droplet. B) 3D schematic of the real‐time phase separation process within an evaporating sessile droplet. C) Brightfield imaging and fluorescence imaging of phase separation between PEG and FITC‐tagged dextran. Scale bar represents 1 mm. D) Schematic of phase separation inside a viral protein‐containing aqueous sessile droplet E) Fluorescence imaging of phase separation between PEG, dextran, and Alexa Fluor® 488 tagged SARS‐CoV‐2 spike protein. The scale bar is 0.5 mm.

An all‐aqueous droplet consisting of PEG and Fluorescein isothiocyanate tagged dextran (FITC‐dextran) was prepared. Upon drying and solvent evaporation, spontaneous phase separation occurred (**Figure** **1**A,B). From the brightfield images in Figure 1C, dispersed small droplets were formed from the edge and merged to the central area (see also Video S1, Supporting Information). Fluorescence images of the same droplet in Figure 1C showed homogenous green fluorescence at time 0 and upon phase separation, enrichment of green fluorescence within the droplets can be observed over time (see also Video S2, Supporting Information). Since the green fluorescence originated only from the FITC‐dextran polymer, we concluded these droplets were dextran‐rich compartments. Alexa Fluor 488 S protein was spiked into the same droplet formulation but using dextran without the fluorescent label (Figure 1D). Figure 1E showed the identical phase separation behavior, in which green fluorescence was evenly distributed at the beginning but spontaneous phase‐separated into tiny droplets at the edge, which finally merged at the central overtime (See also Video S3, Supporting Information). These results confirmed that the spike proteins are localized and concentrated in the dextran‐rich compartments.

### 2.2 Colorimetric Enzyme‐Linked Oligonucleotide Assay (ELONA)

We performed a colorimetric ELONA to quantify the enrichment of spike proteins in the dextran‐rich phase. A 9:1 volume ratio two‐phase mixture was spiked with S protein, and the PEG‐rich and dextran‐rich fractions were extracted separately. By comparing the absorbance intensity quantified from each phase with and without LLPS, S protein concentration was found 11‐fold higher (*p* < 0.05) in DEX‐rich phase and two‐fold lower (*p* < 0.05) in PEG‐rich phase after LLPS (Figure S1, Supporting Information). The results agree with the observations from the real‐time imaging experiments in which S proteins preferentially migrate into the dextran‐rich layer from the PEG‐rich layer. The signal amplification in DEX‐rich phase (11‐fold) also matches the theoretical concentration factor estimated from the volume ratio between two phases (i.e., ten‐fold). This translational experiment further demonstrates the efficacy of spike protein enrichment by LLPS to amplify the detection signal output.

### 2.3 Characterization of Electrochemical Aptamer Biosensor for Spike Protein Detection

**Figure 2:**
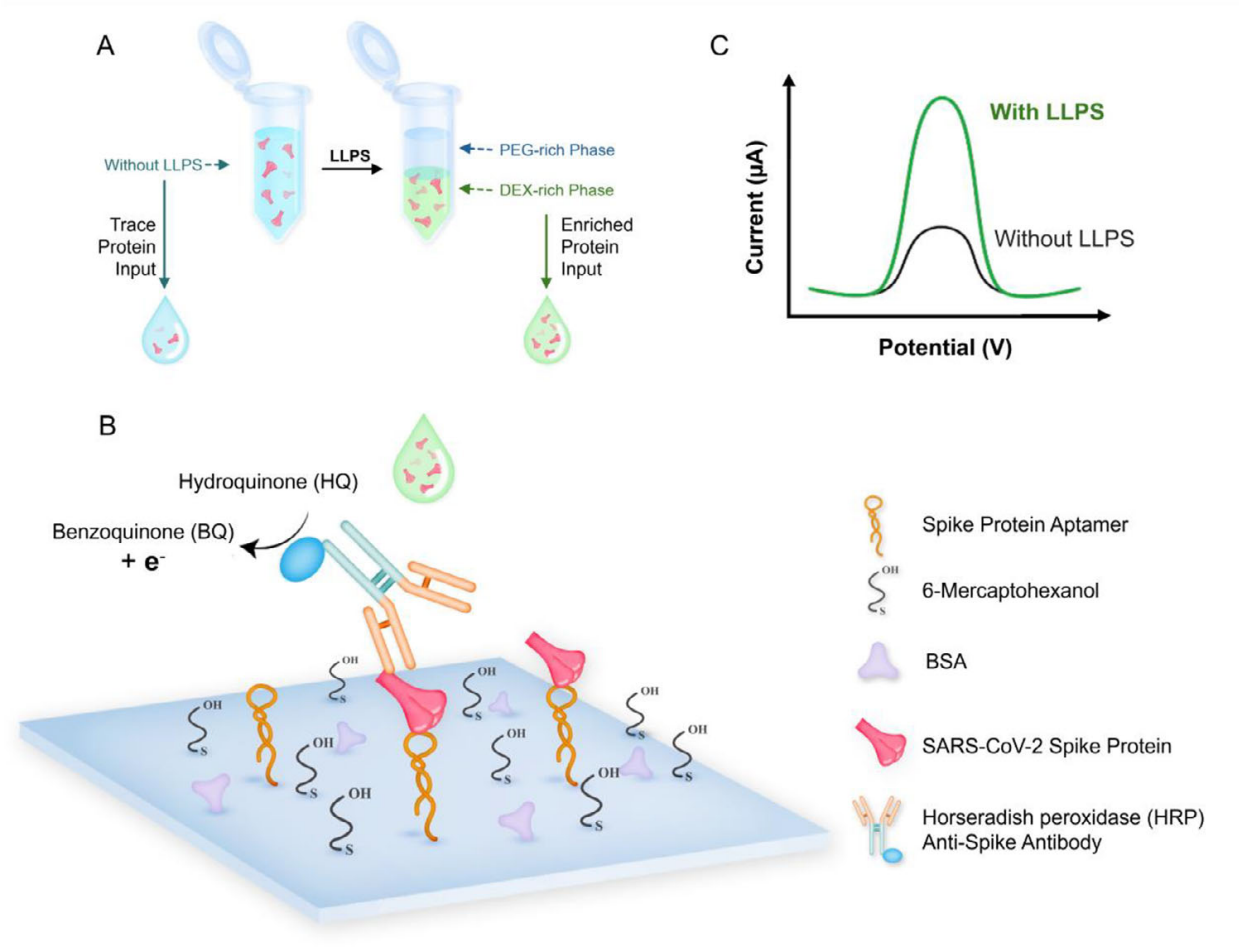
Schematic of LLPS integrated electrochemical aptamer biosensor (E‐AB) for SARS‐CoV‐2 S protein sensing. A) Partitioning of S protein within the dextran‐rich phase in the PEG‐dextran mixture. B) The integration of LLPS samples with enriched S protein into the electrochemical aptamer biosensor (E‐AB). C) Signal outputs of E‐AB with and without LLPS integration.

Electrochemical aptamer biosensors are renowned for their fast signal transduction efficiency and device minimization. LLPS can effectively concentrate the biomarkers from matrices such as saliva, sweat, and urine, where the targets often exist in trace quantity and biosensors without appropriate signal amplification methods may fail to detect their presence.^[^ ^33^, ^35^, ^36^, ^37^, ^38^ ^]^ Therefore, we integrated this segregative LLPS approach with an electrochemical aptamer biosensor to develop a simple and rapid POC diagnostic assay of SARS‐CoV‐2 spike protein (**Figure** **2**).

Detection of spike protein requires sandwich binding between aptamer SNAP1.50,^[^ ^12^ ^]^ spike protein, and anti‐spike protein horseradish peroxidase modified antibody (anti‐Spike HRP‐Ab), followed by the catalytic electrochemical reaction with hydroquinone (HQ) and hydrogen peroxide (H_2_O_2_). Aptamer SNAP1.50 was immobilized on the gold electrode to capture S protein in samples enriched from LLPS. Subsequently, the anti‐Spike HRP‐Ab was incubated to form a sandwich triplex. A series of catalytic reactions occurs as described below when the triplexes are subjected to the substrate (5 mm H_2_O_2_ and 5 mm HQ).^[^ ^39^ ^]^ The signal generation is mediated only when all components are present while the lack of any component resulted in minimal to no signal (Figure S2, Supporting Information).

(1) $$H_{2}O_{2}+\mathrm{HRP}\;\left(\mathrm{reduced}\right)\to \mathrm{HRP}\;\left(\mathrm{oxidized}\right)+H_{2}O$$

(2) $$\mathrm{HRP}\;\left(\mathrm{oxidized}\right)+\mathrm{HQ}\;\to \mathrm{HRP}\;\left(\mathrm{reduced}\right)+\mathrm{BQ}$$

(3)

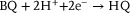




The HRP enzyme is first oxidized by the H_2_O_2_ and then reduced by the presence of HQ. Meanwhile, HQ is oxidized to benzoquinone (BQ). The negative voltage applied to the electrode provides electrons to convert BQ back to HQ. This redox reaction generates a traceable current signal monitored by square wave voltammetry (SWV). The intensity of the signal correlates to the concentration of the sandwich triplex, hence the spike protein in the sample can be quantified.

The spike protein incubation time was optimized to shorten the assay time, which is critical for POC application. S protein samples were incubated with the aptamer immobilized electrode from 0 to 2 h. The assay generated a significantly higher positive signal (*p* < 0.05) when the target protein was incubated for 30 min and reached a plateau after 1 h (Figure S3, Supporting Information). Further incubation resulted in no significant signal improvement. Rapid protein capturing is potentially possible in less than 30 min with further experimental optimizations. We conducted subsequent characterizations with 1 h of target incubation for optimal signal output.

While non‐target proteins may also partition favorably into the dextran‐rich phase, ^[^ ^40^, ^41^ ^]^ the aptamer and antibody bind to their cognate target with high specificity. This was proven by the following specificity study. The E‐AB specificity was tested against potential interference proteins, including the SARS‐CoV‐2 nucleocapsid protein, human immunoglobulin G (IgG), human serum albumin (HSA), and lysozyme. These proteins are either biologically relevant to COVID‐19 infection or naturally exist in human biofluids and may cause potential surface biofouling.^[^ ^42^ ^]^ The S protein produced a substantially higher response compared to all other non‐target proteins, even when presented at much higher concentrations than the target (Figure S4, Supporting Information). This simulates the potential co‐partitioning effect and demonstrates that there is no interference with sandwich E‐AB recognition. The electrode fabrication procedures are sufficient to mitigate non‐specific biofouling by 6‐mercapto‐1‐hexanol (MCH) and bovine serum albumin (BSA) blocking.

**Figure 3:**
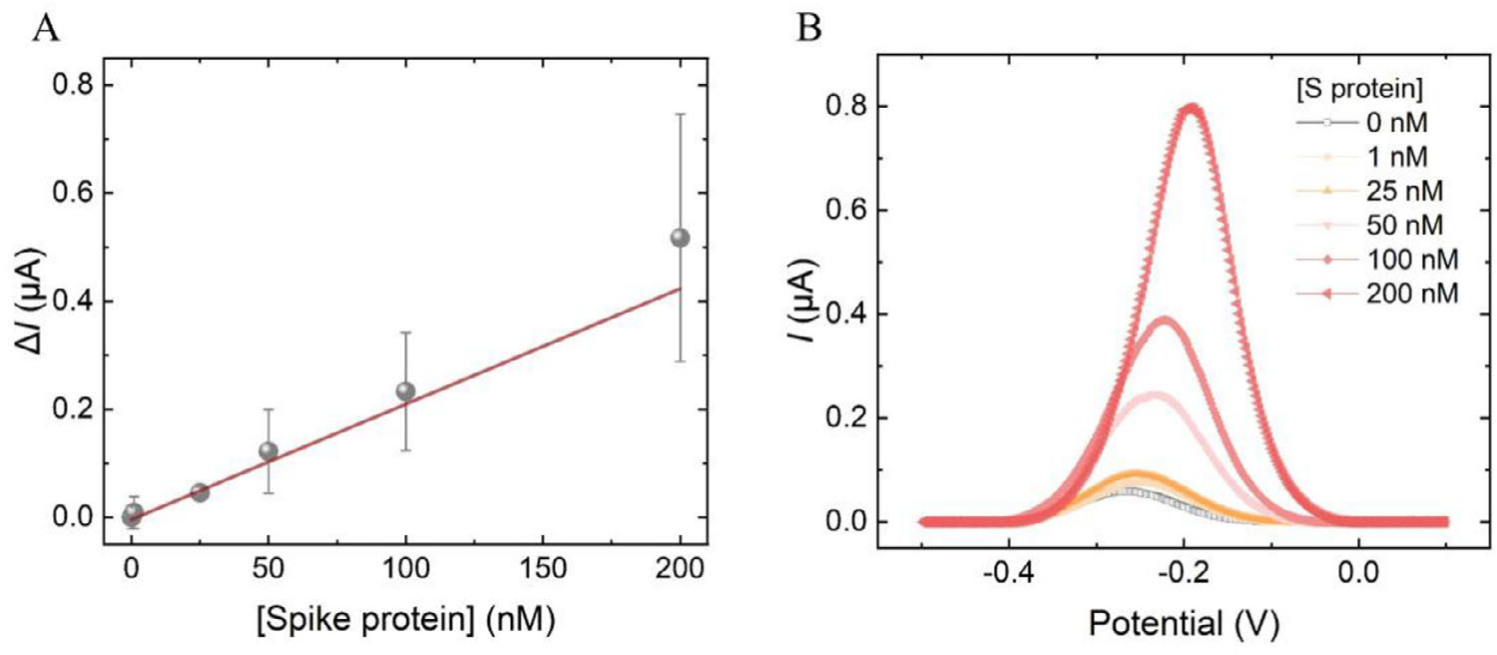
Spike protein detection sensitivity in control PBS buffer without LLPS pre‐enrichment. A) Linear concentration response and B) the corresponding square wave voltammograms. The concentration‐response against S protein was calculated based on the relative current change (Δ*I*) against blank (0 nm target). The error bars represent one standard deviation of uncertainty. Three individual sensors were prepared for each concentration (n = 3).

The sensitivity of the optimized E‐AB prototype against a range of spike protein concentrations without LLPS was then characterized. A linear binding response was observed for spike protein from 1 to 200 nм (**Figure** **3**A). The estimated LOD of the E‐AB without LLPS sample pretreatment is ≈8 nм based on 3.3  ×  S_E_/slope of regression. Overall, our results indicate that the E‐AB prototype can detect S protein with high specificity and sensitivity. The estimated E‐AB sensitivity could potentially be sufficient to detect S protein from patients who are at a severe stage of COVID‐19 infection. However, viral proteins may be present at sub‐nanomolar level in patients during early onset, as inferred from viral load quantification studies.^[^ ^43^, ^44^ ^]^

### 2.4 Integration of LLPS into E‐AB

**Figure 4:**
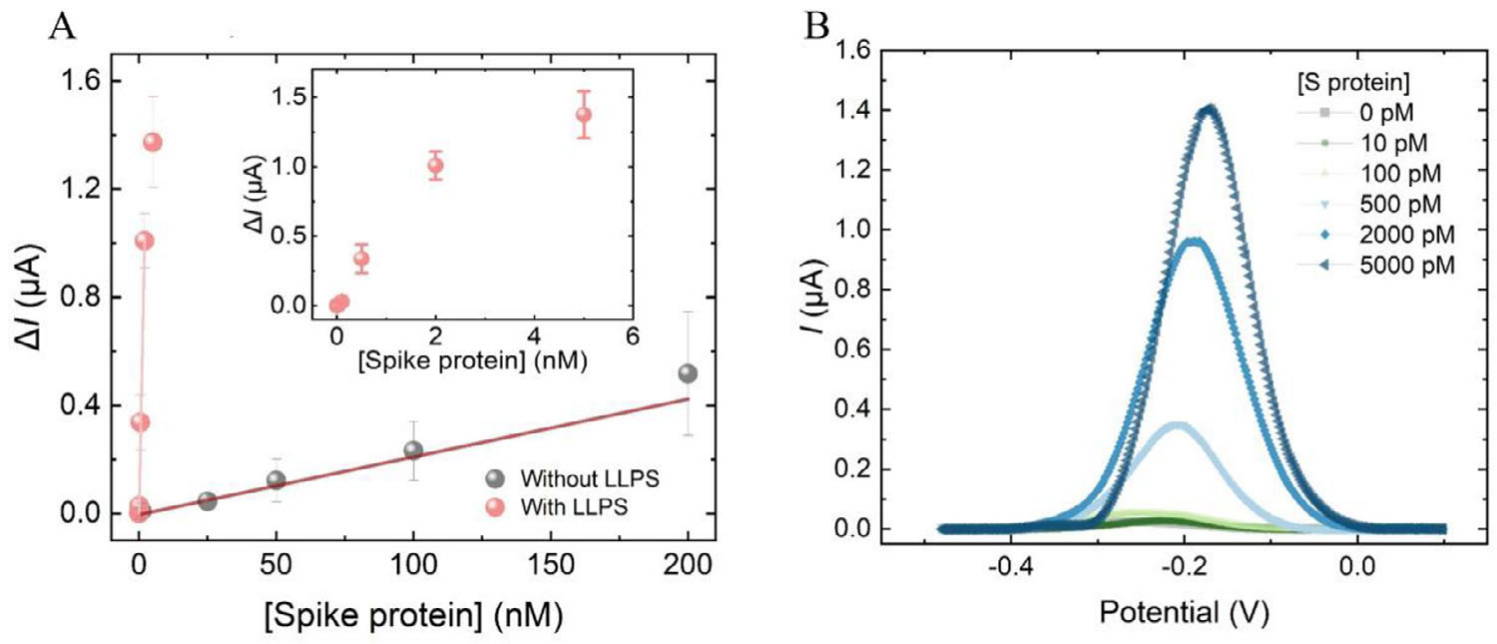
LLPS‐integrated S protein enrichment improves E‐AB detection sensitivity. A) Linear concentration‐response against S protein with and without LLPS enrichment. The concentration response against S protein was calculated based on the relative current change (*ΔI*) against blank (0 nm target). B) Square wave voltammograms of S protein detection with LLPS enrichment. The error bars represent one standard deviation of uncertainty. Three individual sensors were prepared for each concentration (n = 3).

We combined the LLPS into the E‐AB prototype to overcome insufficient clinical sensitivity. We modified the concentration ratio of the two polymers such that upon equilibrium, the volume ratio of PEG‐rich to DEX‐rich aqueous fraction would be 99:1. We thus expected the spike protein would be 100‐fold concentrated in the DEX‐rich fraction. Various concentrations of the S protein were spiked into the LLPS, and the resulting DEX‐rich phase extracts were measured by the E‐AB. As shown in **Figure** **4**A, compared to the sensitivity calibration without LLPS, the pretreatment group exhibits a linear response ranged from 10 pм to 2 nм with an estimated LOD at 55 pм. Compared to the LOD of sensor without pretreatment (8 nм), LLPS enhanced the spike protein detection by 150‐fold. This exceeds the expectation (100‐fold) determined by the theoretical protein enrichment factor according to the designated two‐phase volume ratio. While the increased protein concentration driven by local enrichment remained to be the dominant factor of sensitivity improvement, it is believed that the dextran‐rich phase mimics the intracellular matrix by creating a molecular crowding environment. We have previously proven that such crowding‐induced excluded volume effects boost enzymatic activity governing key cellular activities such as segregative‐associative (SA) droplet formation and ribozyme activity.^[^ ^45^, ^46^ ^]^ The current study further confirms the benefits of LLPS in enzymatic‐based biosensing applications. Although the sensor's dynamic range is a commonly characterized parameter to assess the E‐AB performance, the spike protein level from infected individuals during early stage is expected to be low in the picomolar range.^[^ ^9^, ^47^ ^]^ Therefore, comparing the sensitivity and LoD estimation provides critical evidence on enabling trace protein detection by LLPS integration.

**Figure 5:**
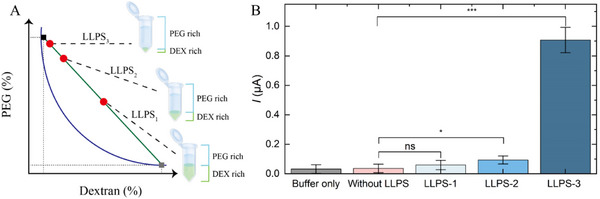
Volume ratio of the two‐phase system dictates the concentration factor into the dextran‐rich layer and thus the E‐AB sensitivity enhancement. A) Simulated phase diagram of PEG/DEX two‐phase system. The blue line is the binodal curve, corresponding to the critical concentrations of the two polymers for spontaneous phase separation. The green line is the tie‐line and the red dots represent typical two‐phase systems with increasing volume ratio (VR) of PEG to DEX layer (VR: LLPS_3_ > LLPS_2_ >LLPS_1_). The intercepts indicate the concentrations of the two polymers in the top PEG layer (Black rectangle) and the bottom DEX layer (Gray rectangle) when a two‐phase system is formed along the tie‐line (i.e., LLPS_1/2/3_). B) Comparison of S protein detection signal (in terms of current; *I*) enriched from the three‐volume ratios of LLPS. “Buffer only” refers to dextran‐rich phase extracted from the LLPS without spiking S protein (without protein). The error bars represent one standard deviation of uncertainty. Three individual sensors were prepared for each concentration (n = 3). ns denotes non‐significant, *p* > 0.05. * denotes *p* ≤ 0.05. *** denotes *p* ≤ 0.001

Next, we studied the effect of altering the volume ratio on the E‐AB signaling improvement. From the phase diagram shown in **Figure** **5**A, LLPS of different volume ratios (red dots) can be prepared by selecting compositions on the same tie‐line (green line). Since the intercepts (black and grey rectangles) between the tie‐line and the binodal curve (blue curve) determine the polymeric concentrations in the two phases upon phase separation, all LLPS systems formed along the tie‐line share the same S protein partition coefficient.^[^ ^26^ ^]^ Therefore, we anticipated the more extreme the volume ratio, the better the concentration effect leading to increased E‐AB detection sensitivity. This hypothesis was verified by enriching S protein in LLPS of different volume ratios (1:1, 9:1, and 99:1 from LLPS_1_, LLPS_2_, and LLPS_3_, respectively), performing the E‐AB detection assay along with buffer only and no LLPS controls (Figure 5B). S protein extracted from LLPS_1_ did not generate a significant signal gain compared to no enrichment control (*p* = 0.2335). On the other hand, samples extracted from LLPS_2_ and LLPS_3_ showed evident improvement (*p* = 0.0084 and *p* < 0.0001, respectively). This can be explained by an insufficient degree fold concentration of LLPS_1_ resulting in S protein in the sample remaining undetected. Through enrichment by 10‐ and 100‐fold in LLPS_2_ and LLPS_3_, S protein can be significantly concentrated and reliably detected by the E‐AB at a concentration otherwise undetectable without the sample pre‐treatment through segregative LLPS.

Lastly, we tested the compatibility of LLPS to enrich the S protein from human biofluids. Apart from nasopharyngeal and oropharyngeal swab sampling methods, collection of drooling saliva or urine are preferred as they are easily accessible in large quantities and cause minimal patient discomfort whereas human serum provides a minimally invasive sampling method to unlock the systemic molecular landscape for disease diagnosis. To verify the efficacy of LLPS in such biofluids, S protein was spiked into 99:1 VR of LLPS prepared in either 100% artificial saliva, 100% synthetic urine and 1% human serum compared to 1 × PBS. The enriched S protein extracts were subjected to E‐AB. Saliva, urine and serum‐based LLPS all demonstrated successful protein enrichment evidenced by the obvious gain in electrochemical signal, whereas the protein without enrichment remained undetected (Figure S5, Supporting Information). This shows the potential applicability of the two‐phase system in these biofluids. While matrix effects such as saliva viscosity or the presence of a wide range of non‐target protein in human samples may present translational challenges, several studies have demonstrated compatible detection in undiluted or minimally processed biofluids using electrochemical aptamer biosensors.^[^ ^16^, ^48^, ^49^, ^50^ ^]^ These findings support the feasibility of our LLPS E‐AB in clinical applications. This study potentially advances the development of POC diagnostics by overcoming challenges of sensitivity associated with low target analyte concentrations.

## 3 Conclusion

In this paper, we reported the application of a novel all‐aqueous phase segregation approach to improve the electrochemical aptamer biosensor performance by analyte enrichment and molecular crowding effect. We provide an alternative and promising solution to improve assay sensitivity. Using the SARS‐CoV‐2 spike protein and Aptamer SNAP1.50 as a model, we demonstrated predictable enrichment by LLPS at designated volume ratio, integrating the approach with an aptamer electrochemical biosensor. Unlike other signal amplification strategies, LLPS is unique and simple, focusing on upstream protein enrichment from saliva and other biofluids. The system is flexible to enrich other proteins by optimizing the composition of the two polymers. This is especially applicable to viral proteins that rely on biological LLPS to concentrate for viral replication in the host cell.^[^ ^51^, ^52^ ^]^ Similarly, other aptamers could be implemented for alternative target‐specific detection. Therefore, the prototype holds broad potential for diagnostic measurement of various protein and other biomarker analytes in POC settings through application specific customization.

## 4 Experimental Section

### Materials and Instruments

All oligonucleotides were order from Sangon (China). Thin film gold microelectrodes (ED‐SE1‐AuPt) were purchased from MicruX Technologies (Spain). SARS‐CoV‐2 spike protein (S1N‐C52H3) was purchased from ACROBiosystem. Recombinant SARS‐CoV‐2 S GCN4‐IZ Alexa Fluo 488 Protein (AFG10796) was purchased from Bio‐Techne. Horseradish peroxidase conjugated recombinant anti‐SARS‐CoV‐2 spike glycoprotein antibody (anti‐Spike HRP‐Ab) was purchased from Abcam. Dextran (M.w. = 10 kDa) was purchased from Aladin. Lysozyme, human serum immunoglobulin (IgG), human serum albumin (HSA), human serum (H4522), artificial saliva, synthetic urine diluent, 30% hydrogen peroxide (H_2_O_2_), hydroquinone (HQ), 6‐Mercapto‐1‐hexanol (MCH), Tris(2‐carboxyethyl)phosphine (TCEP), Poly(ethylene glycol) (PEG, BioUltra, M.w. = 8 kDa) and Fluorescein isothiocyanate‐dextran (M.w. = 4 kDa) were purchased from Sigma. All electrochemical measurements were conducted using PalmsSens4 potentiostat with Drop‐cell Connector (ED‐DROP‐CELL) purchased from MicruX technologies.

### Verification of LLPS Spike Protein Concentration by Microscopic Imaging Assay

Blank aqueous droplets were prepared by PEG and FITC‐dextran. Spike protein‐containing droplets were prepared by PEG, dextran, and Alexa Fluor 488 recombinant S protein. Both droplet formulations were prepared at concentrations of 9 wt.% PEG and 4 wt.% dextran to form all‐aqueous droplets. The droplets were pipetted onto clean microscope glass slides (ISOLAB GmbH) at room conditions (22 °C, 55–65% humidity). Slides were prepared by wiping with ethanol, sonicating in ethanol for 15 min, washing with isopropyl alcohol and Milli‐Q water, and drying with nitrogen flow before heating at 65 °C for 1 h. Brightfield images and fluorescence images were captured using a Nikon Ti2‐E Widefield microscope with a 475 nm excitation wavelength for both FITC‐dextran and Alexa Fluor 488 channels. Image processing was performed using ImageJ (NIH) software.

### Colorimetric Enzyme‐Linked Oligonucleotide Assay (ELONA)

Biotinylated SNAP1.50 DNA aptamer was diluted to 1 µм in PBSMT (1× PBS with 2 mм MgCl_2_ and 0.05% Tween‐20). 100 µL of diluted aptamer solution was added to Pierce Streptavidin Coated High‐Capacity Plate (ThermoFisher) per well and incubated overnight with shaking at 4 °C. Then, the plate was washed three times in PBSMT. 5% (w/v) bovine serum albumin (BSA) solution (GoldBio) in PBSMT was added for surface blocking by incubating for at least 2 h, followed by PBSMT washing. 100 µL of spike protein standards (10 nм) or samples extracted from the two‐phase equilibrium mixture (after centrifugation at 3000 × g for 5 min) was added and allowed to bind for 1 h. After washing, anti‐Spike HRP‐Ab (Abcam, 1:5000 dilution in PBST) was added to bind for 30 min. After washing with PBSMT, 50 µL of 1‐Step Ultra TMB‐ELISA Substrate (ThermoFisher) was added and incubated for 15 min. All steps mentioned above were processed at room temperature unless otherwise noted. H_2_SO_4_ (2 m) was added to quench the reaction. The absorbance at 450 nm was measured by Varioskan Flash spectral scanning multimode reader (ThermoFisher).

### Electrochemical Aptamer Sensor Fabrication

The MicruX thin film electrodes were first rinsed with ethanol and then with Milli‐Q water. The electrodes were sonicated in a water bath for 5 min and further cleaned by cyclic voltammetry (CV) in 0.5 м NaOH from −0.35 to −1.35 V for 50 cycles. Followed by CV sweep in 0.5 м H_2_SO_4_ from −0.35 to 1.25 V at 0.1 V s^−1^ for 10 cycles.

Thiol‐modified SNAP1.50 aptamers were first reduced by TCEP (oligo to TCEP molar ratio was 1:100) for 2 h at room temperature to cleave the disulfide bonds. The reduced aptamer solution was diluted in 1×PBS to 0.5 µм. Five microliters of the solution was dropped on the gold for immobilization overnight at room temperature. After thorough rinsing with Milli‐Q, the electrodes were blocked with 6 mм MCH for 3 h. The electrodes were rinsed and further blocked with 1% BSA solution for 1 h. All incubation steps involved humidity control to prevent solution drying. The electrodes were rinsed and stored at 4 °C before use.

### LLPS Concentration and Electrochemical Measurement

The LLPS was first prepared by dissolving and mixing PEG (average M.w. = 8 kDa) and dextran T10 (average M.w. = 10 kDa) in buffer. Spike protein was added and the LLPS was homogenized and centrifuged for 5 min at 3000 × g to collect the concentrated spike protein from the bottom DEX‐rich phase. The extracted DEX‐rich phase was dropped onto an aptamer immobilized gold electrode for 1 h at RT for binding. After thorough rinsing with PBS, 10 nм of the anti‐Spike HRP‐Ab solution was added to incubate for 45 min. Finally, a substrate solution (5 mм H_2_O_2_ and 5 mм HQ in 1×PBS) was added for 5 min of incubation. The electrodes were immediately interrogated by square wave voltammetry (SWV) from −0.5 to 0.1 V. The measuring amplitude and frequency were 5 mV and 25 Hz respectively. The peak current values at ≈−0.2 V were recorded as the signal output. Square wave voltammograms were extracted from PSTrace 5.9.

### Statistical Analysis

The error bars represent one standard deviation of uncertainty. Unless specified, three individual sensors were prepared for each concentration (n = 3). Two‐sample t tests were conducted to determine the statistical significance between any of the two experimental groups. ns denotes non‐significant, *p* > 0.05. * denotes *p* ≤ 0.05. *** denotes *p* ≤ 0.001. All data were analyzed and plotted by Origin.

## Conflict of Interest

The authors declare no conflict of interest.

## Supporting information



Supporting Information

Supplemental Video 1

Supplemental Video 2

Supplemental Video 3

## Data Availability

The data that support the findings of this study are available in the supplementary material of this article.
