# Supplementary material for: All‐Aqueous Phase Segregation Integrated Electrochemical Aptamer Biosensor Enables Picomolar Detection of SARS‐CoV‐2 Spike Protein
Source: Small. 2025 Oct 3;21(45):e03466. doi: 10.1002/smll.202503466 (PMC12614154; doi:10.1002/smll.202503466)
Supplement: Supplementary file 1 — Supporting Information [file SMLL-21-e03466-s001.docx]

Supporting Information

All-aqueous Phase Segregation Integrated Electrochemical Aptamer Biosensor Enables Picomolar Detection of SARS-CoV-2 Spike Protein

Ryan Ho-Ping Siu^†^, Yage Zhang^†^, Sihan Liu, Andrew Brian Kinghorn, Wei Guo,* Ho Cheung Shum,* Julian Alexander Tanner*

^†^These authors contributed equally to this work.

*Corresponding authors: [weiguo@szu.edu.cn](mailto:weiguo@szu.edu.cn); [ashum@cityu.edu.hk](mailto:ashum@cityu.edu.hk); [jatanner@hku.hk](mailto:jatanner@hku.hk).


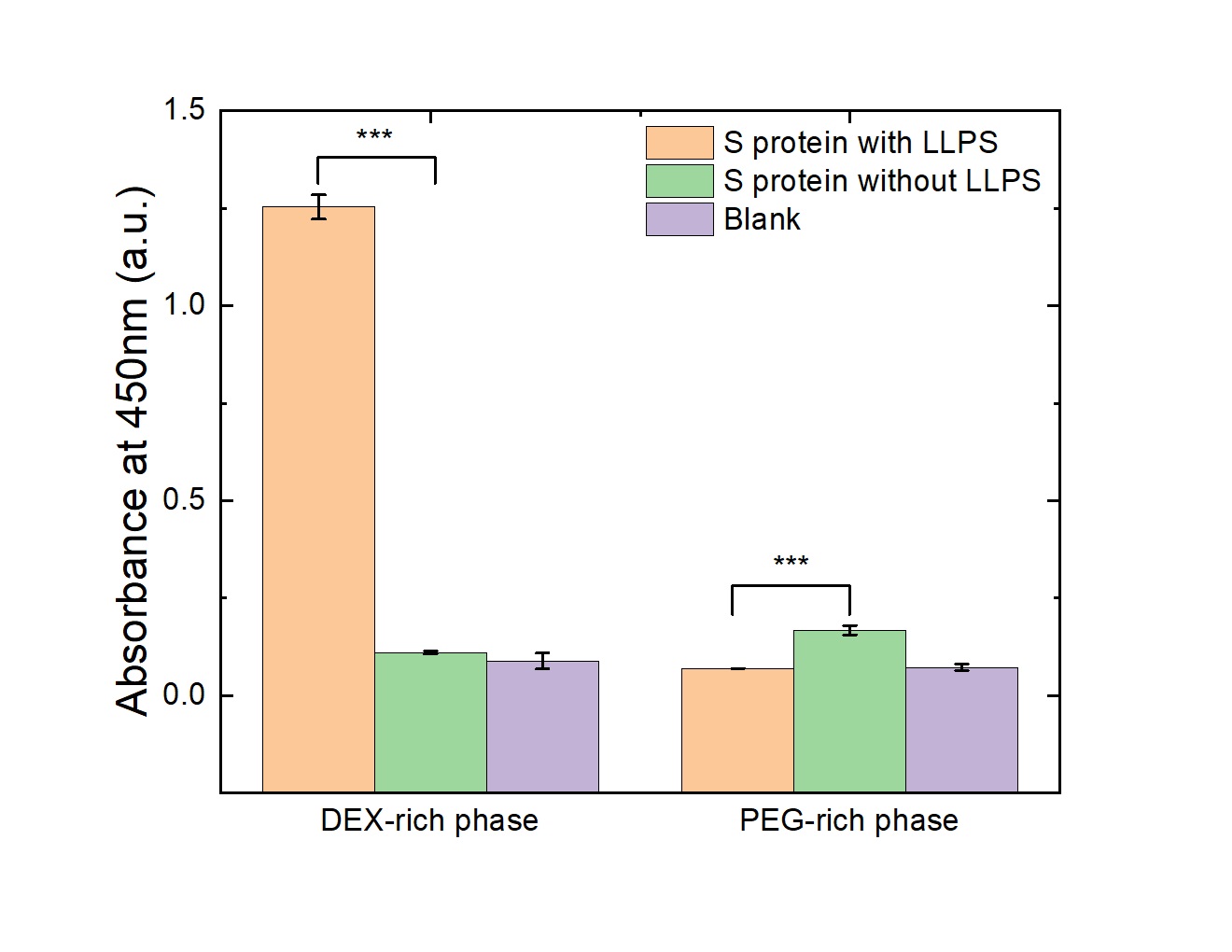


**Figure S1. Verification of SARS-CoV-2 spike (S) protein partitioning in LLPS by colorimetric ELONA.** S protein spiked into LLPS was phase-separated into dextran-rich and PEG-rich phases. The concentration of S protein in each phase was quantified by colorimetric ELONA using aptamer SNAP1.50 and HRP-anti spike antibody. S protein preferential concentration can be verified as the colorimetric intensity increase in DEX-rich phase, along with the decrease in intensity in PEG-rich phase after LLPS. The error bars represent one standard deviation of uncertainty. (n = 3)


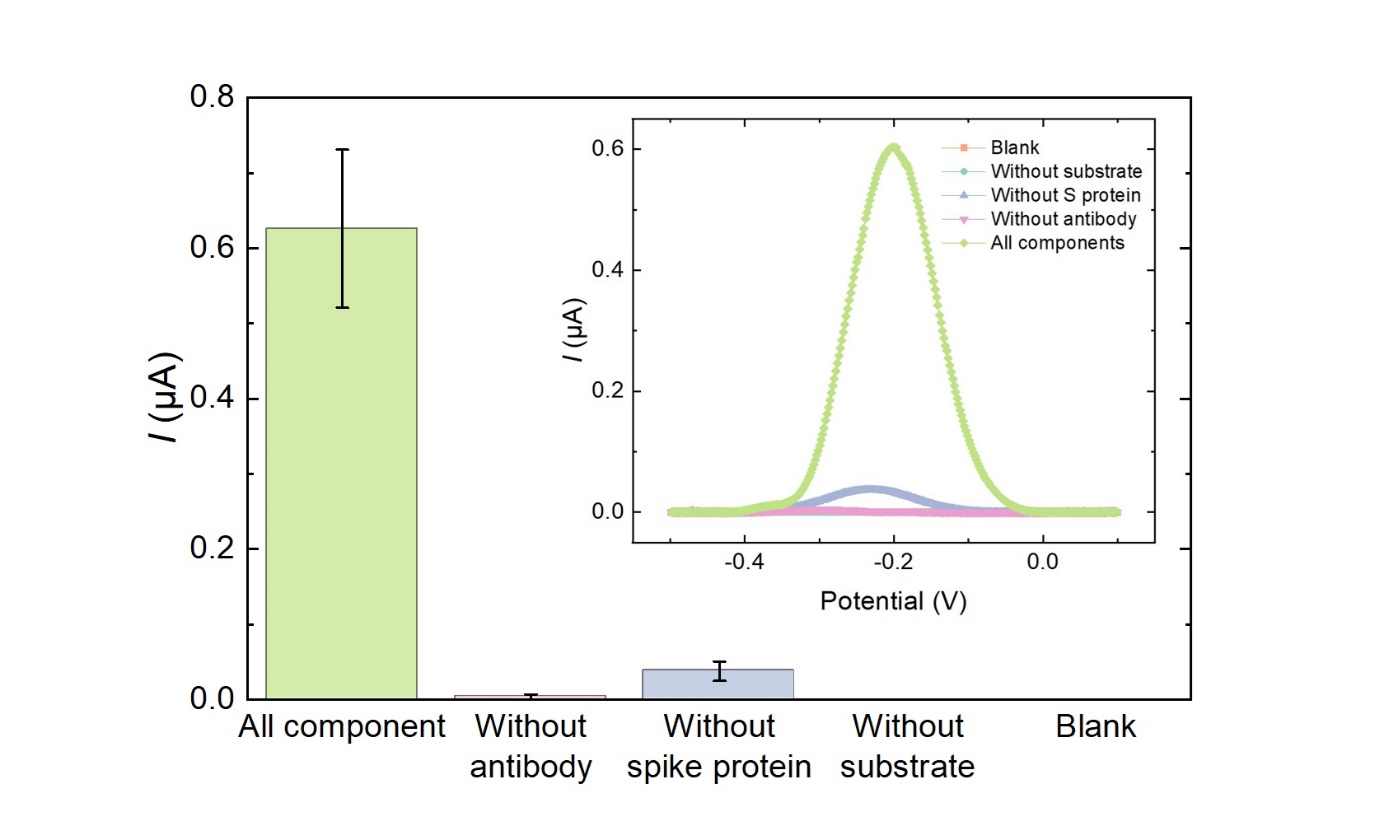


**Figure S2. Feasibility of spike protein sensing by the sandwich E-AB assay.** Electrochemical sensing is possible only in the presence of SNAP1.50 aptamer, spike protein, anti-Spike HRP-Ab and the substrate solution. The error bars represent one standard deviation of uncertainty. (n = 3)


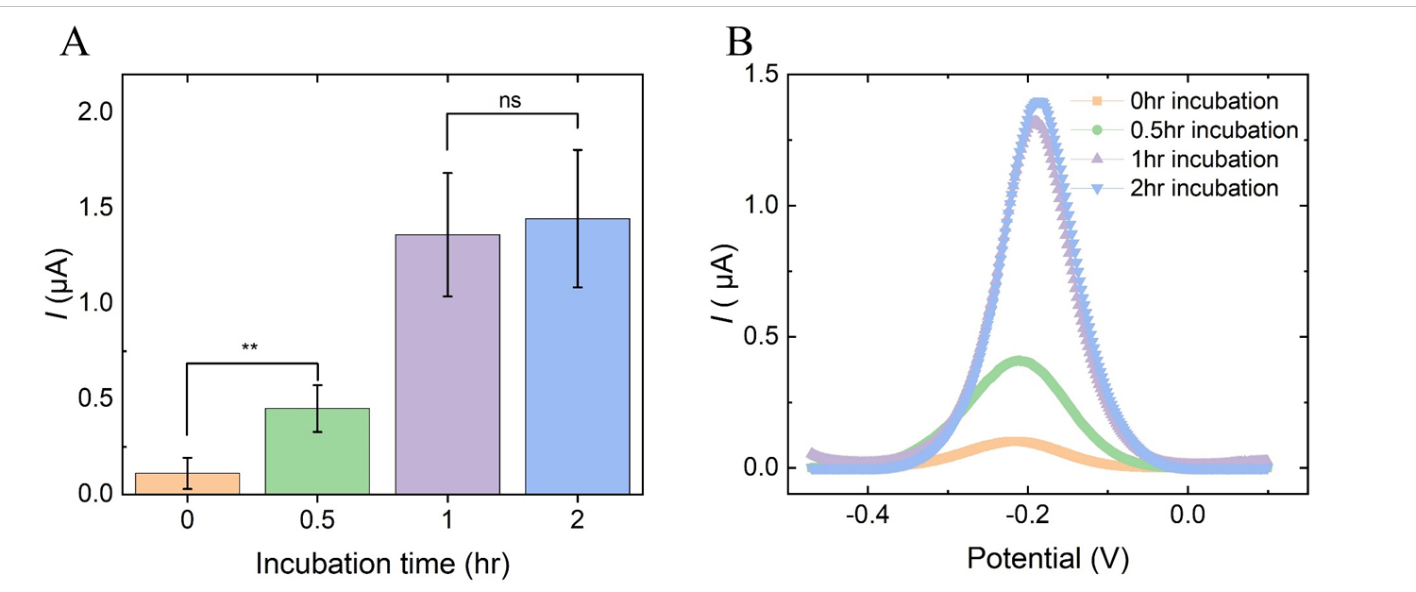


**Figure S3. Optimization of spike protein incubation time.** A) The average E-AB signals with increasing sample incubation time and B) the corresponding square wave voltammograms. Positive S protein sensing requires at least 30 minutes of incubation on the aptamer immobilized gold electrode. Signal plateaus after 1 hour incubation. The error bars represent one standard deviation of uncertainty. (n = 3)


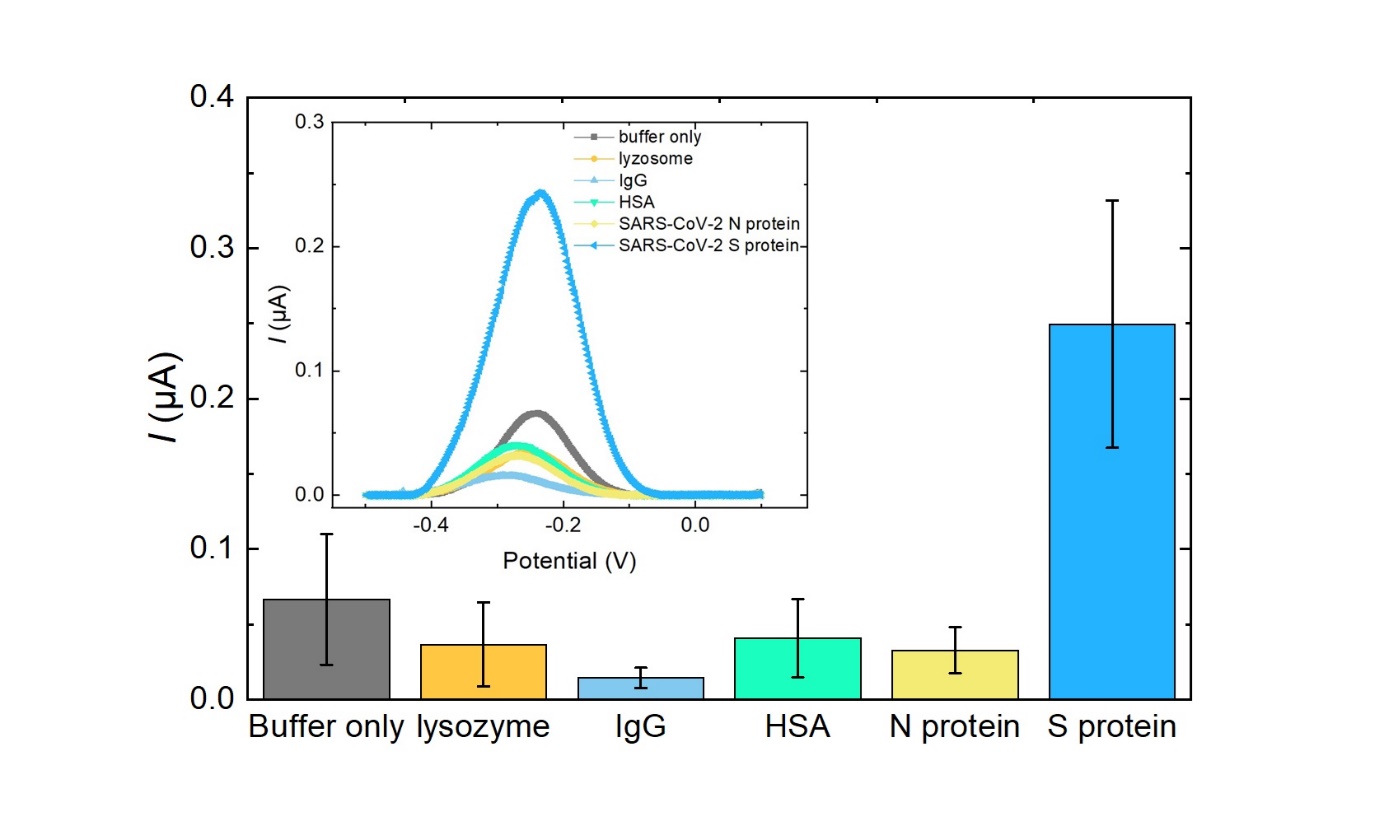


**Figure S4. Specificity of the Spike protein sensing E-AB.** The protein concentration applied was 200 nM for SARS-CoV-2 nucleocapsid (N) and spike (S) protein or 1 μM for lysozyme, human IgG and HSA. The error bars represent one standard deviation of uncertainty. (n = 3)


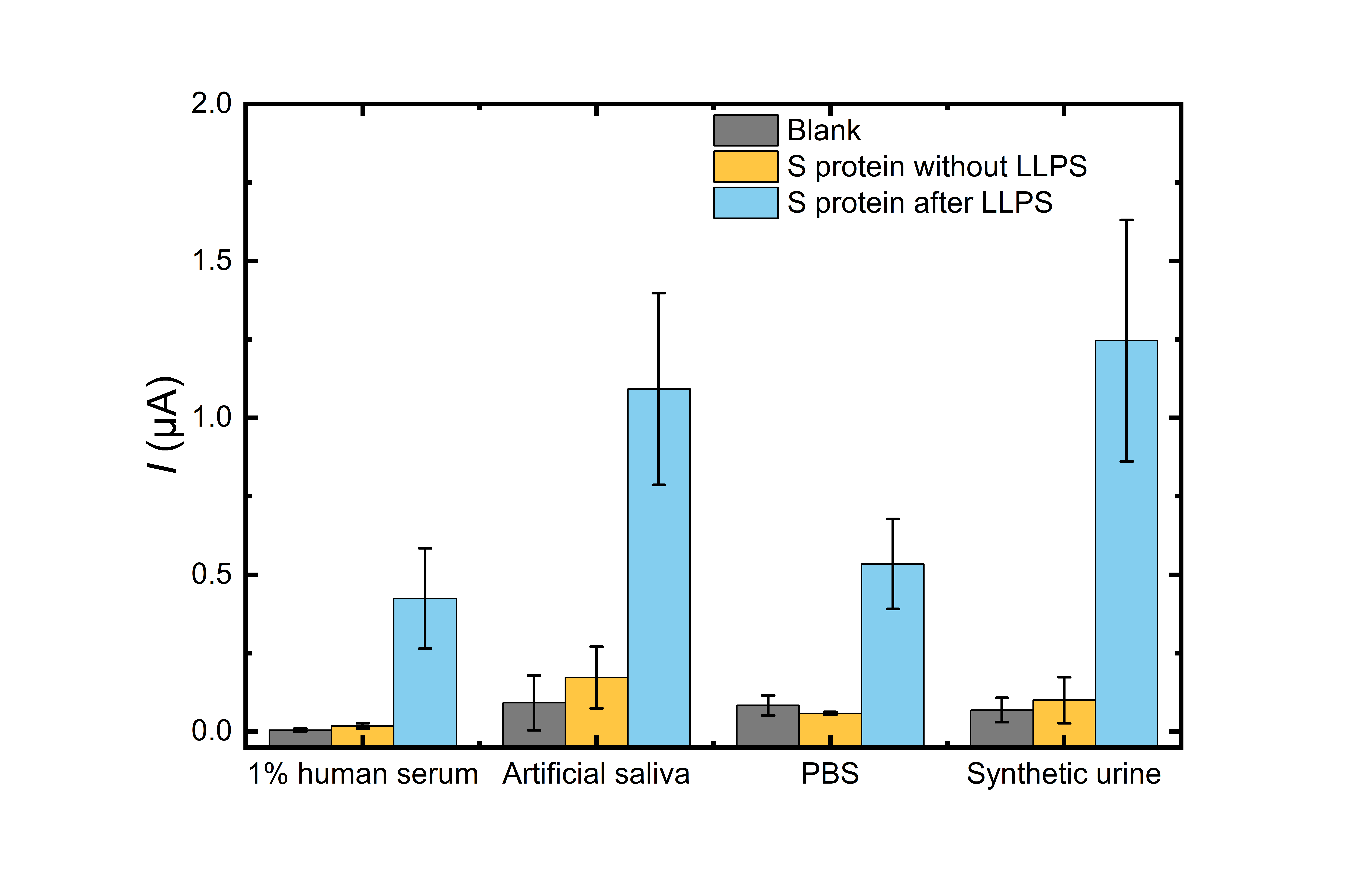


**Figure S5. Compatibility of spike protein concentration in human biofluids by LLPS.** The two-phase system (99:1 volume ratio of PEG-rich to DEX-rich phase) was prepared in either control PBS, 100% artificial saliva, 100% synthetic urine or 1% human serum. S protein enrichment by LLPS is independent to the human biofluid composition. The error bars represent one standard deviation of uncertainty. (n = 3)
